# Roles and Mechanisms of Astragaloside IV in Combating Neuronal Aging

**DOI:** 10.14336/AD.2022.0126

**Published:** 2022-12-01

**Authors:** Qumar Zaman, Dahong Zhang, Obireddy Sreekanth Reddy, Wing-Tak Wong, Wing-Fu Lai

**Affiliations:** ^1^Department of Urology, Zhejiang Provincial People's Hospital, Affiliated People's Hospital, Hangzhou Medical College, Hangzhou 310000, China.; ^2^Department of Applied Biology and Chemical Technology, Hong Kong Polytechnic University, Hong Kong Special Administrative Region, China.; ^3^Department of Chemistry, Sri Krishnadevaraya University, Anantapur 515003, India.; ^3^Ciechanover Institute of Precision and Regenerative Medicine, The Chinese University of Hong Kong (Shenzhen), Shenzhen 518172, China,

**Keywords:** Neuronal aging, astragaloside IV, neurodegeneration, mitochondrial dysfunction, energy-sensing pathways

## Abstract

Aging can lead to changes in the cellular milieu of the brain. These changes may exacerbate, resulting in pathological phenomena (including impaired bioenergetics, aberrant neurotransmission, compromised resilience and neuroplasticity, mitochondrial dysfunction, and the generation of free radicals) and the onset of neurodegenerative diseases. Furthermore, alterations in the energy-sensing pathways can accelerate neuronal aging but the exact mechanism of neural aging is still elusive. In recent decades, the use of plant-derived compounds, including astragaloside IV, to treat neuronal aging and its associated diseases has been extensively investigated. This article presents the current understanding of the roles and mechanisms of astragaloside IV in combating neuronal aging. The ability of the agent to suppress oxidative stress, to attenuate inflammatory responses and to maintain mitochondrial integrity will be discussed. Important challenges to be tacked for further development of astragaloside IV-based pharmacophores will be highlighted for future research.

## 1.Introduction

Neurons endure various stresses led by the accumulation of structurally or functionally impaired proteins, resulting in disruption of the integrity of the plasma membrane and genome. These stresses subsequently deteriorate the functions of neurons, induce apoptosis, and promote neuronal aging and its associated hallmarks [1], including mitochondrial impairments [2], synaptic degeneration [3], dysregulated Ca^2+^ levels [4], changes in energy-sensing pathways [5], and enhanced oxidative stress [6]. Because neural senescence promotes anatomical pathologies (e.g., white matter lesions and brain atrophy) [7-9], neuronal aging at the end compromises the functional capacity of brain and various physiological processes (including blood supply) to escalate brain aging [10]. Right now, our understanding of the process of neuronal aging is still limited, but recent efforts devoted to exploring pathophysiological processes underlying neurological diseases have enabled the identification of various potential therapeutic strategies to combat neuronal aging [11, 12]. For example, the activation of the antioxidant response element (ARE) cascade has been found to lead to the up-regulation of the expression of *Nrf2* and other ARE-associated genes, including the oxygenase-1 (*HO-1*) gene, to mitigate the neural damage [13, 14]. This paves the way for combating neuronal aging in practice.

**Figure 1.**
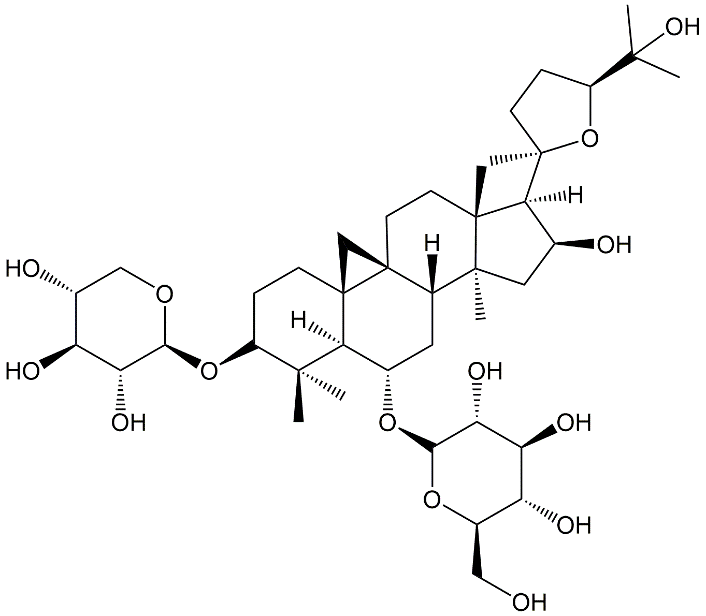
Chemical structure of astragaloside IV.

**Figure 2.**
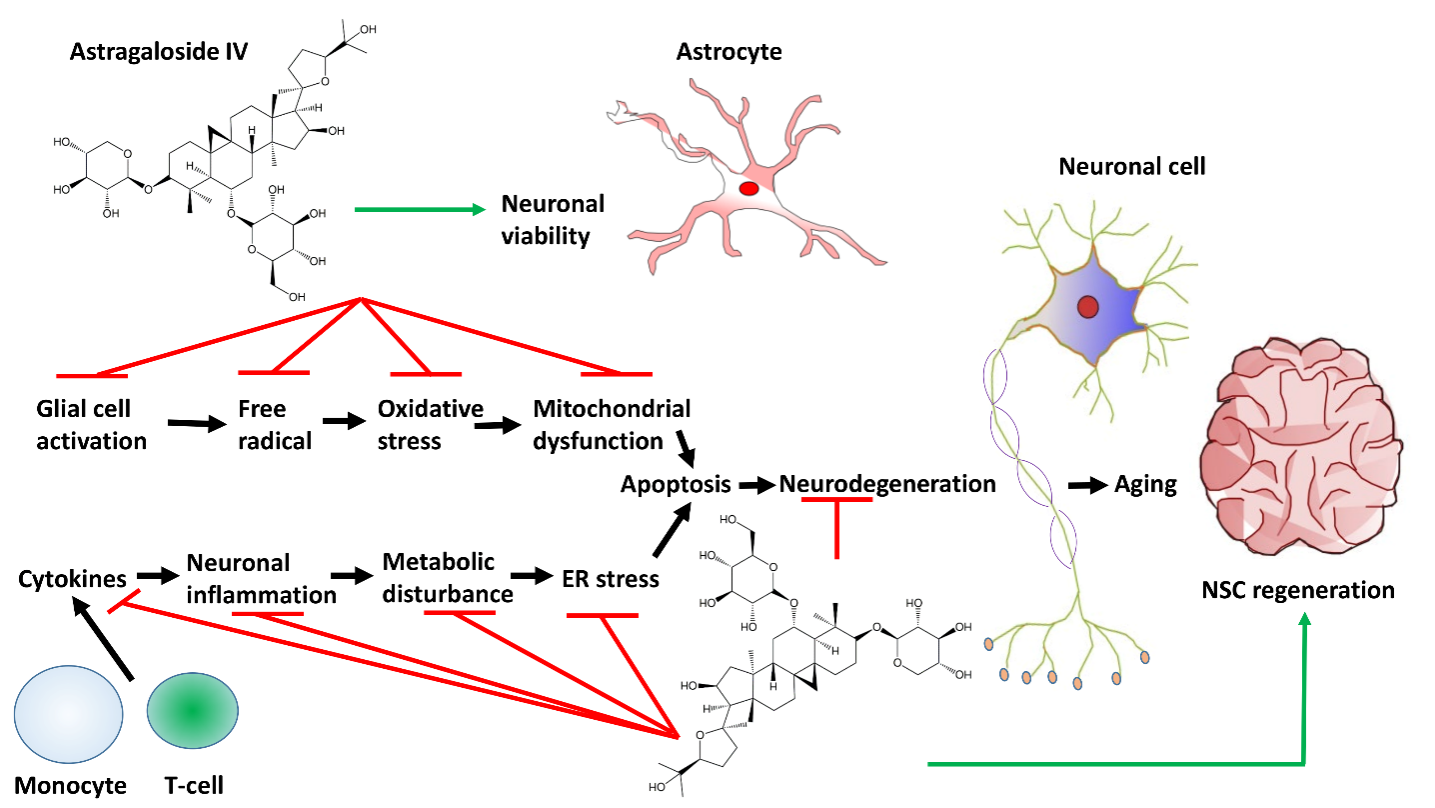
A schematic diagram illustrating the use of astragaloside IV to combat neuronal aging and related disorders. Red lines represent inhibition; whereas green lines represent promotion.

Among different strategies exploited, the use of medicinal herbs to treat neurological aging and its associated diseases has gained increasing attention from the scientific community (Table 1) [15-31]. Astragaloside IV is one of the botanical compounds possessing multi-target therapeutic properties. Astragaloside IV (also known as 3-O-β-D-xylopyranosyl-6-O-β-D-gluco-pyranosyl-cycloastragenol) has the molecular formula of C_14_H_68_O_14_. It is a highly polar tetracyclic triterpenoid saponin (Fig. 1) [32] and has been characterized as a potential therapeutic agent to tackle different neurodegenerative disorders (including motor deficits and aberrant neurotransmission) due to its strong capability to counteract oxidative stress and inflammatory responses [33]. Despite its therapeutic potential, poor oral bioavailability [34] and poor aqueous solubility [35] are some of the major hurdles to be overcome during the development of astragaloside IV-based therapeutic agents. The focus of this review is to provide an overview of recent research on the roles and mechanisms of astragaloside IV as a pharmacologic agent to tackle neuronal aging (Fig. 2). Research gaps and potential challenges for the development of astragaloside IV- based interventions will also be discussed for future research.

**Table 1:** Examples of plant-derived compounds that have been reported to ameliorate neuronal aging.

Compound	Source	Effects	Ref.
Epigallocatechin-3-gallate	*Camellia sinensis*	Protecting mitochondria in the brain against oxidative damage	15
		Suppressing cognitive decline, brain atrophy and oxidative damage	16
		Eliciting antioxidative and anti-inflammatory effects	17
Astragaloside IV	*Astragalus membranaceus*	Restoring the telomere length in neurons	18
Gastrodin	*Gastrodia elata*	Suppressing microglial activation and restoring neurotransmission	19
Trolox	*Punica granatum* L.	Protecting hippocampal neurons and improving memory	20
Taxifolin	*Taxus sumatrana*	Inhibiting the development of β-amyloid	21
Kaempferol	*Mespilus germanica* L.	Reducing neuroinflammation	22
Piceatannol	*Vitis vinifera*	Ameliorating neuronal hippocampal pathology	23
Ligstroside	*Olive cultivars*	Improving the bioenergetics of mitochondria	24
Plumbagin	*Juglans regia*	Improving cognitive function	25
Arctigenin	A*rctium lappa* L.	Promoting neuronal survival and function	26
Tyrosine	Sesamum indicum	Rescuing fronto-striatal activation in an age-dependent manner	27
Myricetin	*Vaccinium subg. oxycoccus*	Attenuating brain injury and neurological deficits	28
Tannins	*Schinopsis balansae*	Eliciting antioxidative and anti-inflammatory effects	29
Quercetin	*Allium cepa*	Alleviating neuroinflammation	30
Butein	*Rhus lancea*	Alleviating neuroinflammation and oxidative stress	31

## 2. Effects of astragaloside IV on amelioration of neuronal aging

Astragaloside IV has played multiple roles in combating neuronal aging. For instance, along with notoginsenoside R1, ginsenoside Rb1 and ginsenoside Rg1, it has been reported to enhance nerve cell survival by decreasing the levels of nitric oxide and malondialdehyde (MDA) while promoting the expression of superoxide dismutase (SOD) [36]. It has also been shown to inhibit brain damage caused by subarachnoid hemorrhage (SAH) [37], which leads to a decline in the activity of glutathione peroxidase (GSH-Px) and superoxide dismutase and accelerates apoptosis in neurons. Astragaloside IV can alleviate oxidative stress and improve the neurobehavioral outcome in mice suffering from SAH by inhibiting the expression of *IL-1β, IL-6*, and *TNF-α* and by promoting the up-regulation of GSH-Px, catalase (CAT) and SOD [38]. Apart from this, by promoting the density of the myelinated fibre and by elevating the level of glutathione peroxidase [39], astragaloside IV can enhance the motor nerve conduction velocity (MNCV) in rats. It can also improve the learning and memory in rats suffering from chronic cerebral hypoperfusion by increasing the level of SOD and by attenuating lipid peroxidation, DNA damage, and apoptosis in the hippocampus [40].

More recently, astragaloside IV has been reported to inhibit apoptosis and alleviate the reactive oxygen species (ROS) generation in human neuronal cells by up-regulating the expression of tyrosine hydroxylase and α-synuclein and by inhibiting *Bax* expression [41]. It has also promoted the mitochondrial membrane potential and has attenuated oxidative stress in retinal neurons by down-regulating *CASP3* expression [42]. Astragaloside IV, therefore, shows the capability of regenerating intercellular connections and inhibiting ROS generation via its effect on oxidative stress [43]. Apart from alleviating oxidative stress, astragaloside IV can help combat neuronal aging via multiple mechanisms, ranging from modulation of neuroinflammation to enhancement of mitochondrial integrity. This will be discussed in the following parts of this section.

### 2.1. Combating neuroinflammation and glial cell activation

**Figure 3.**
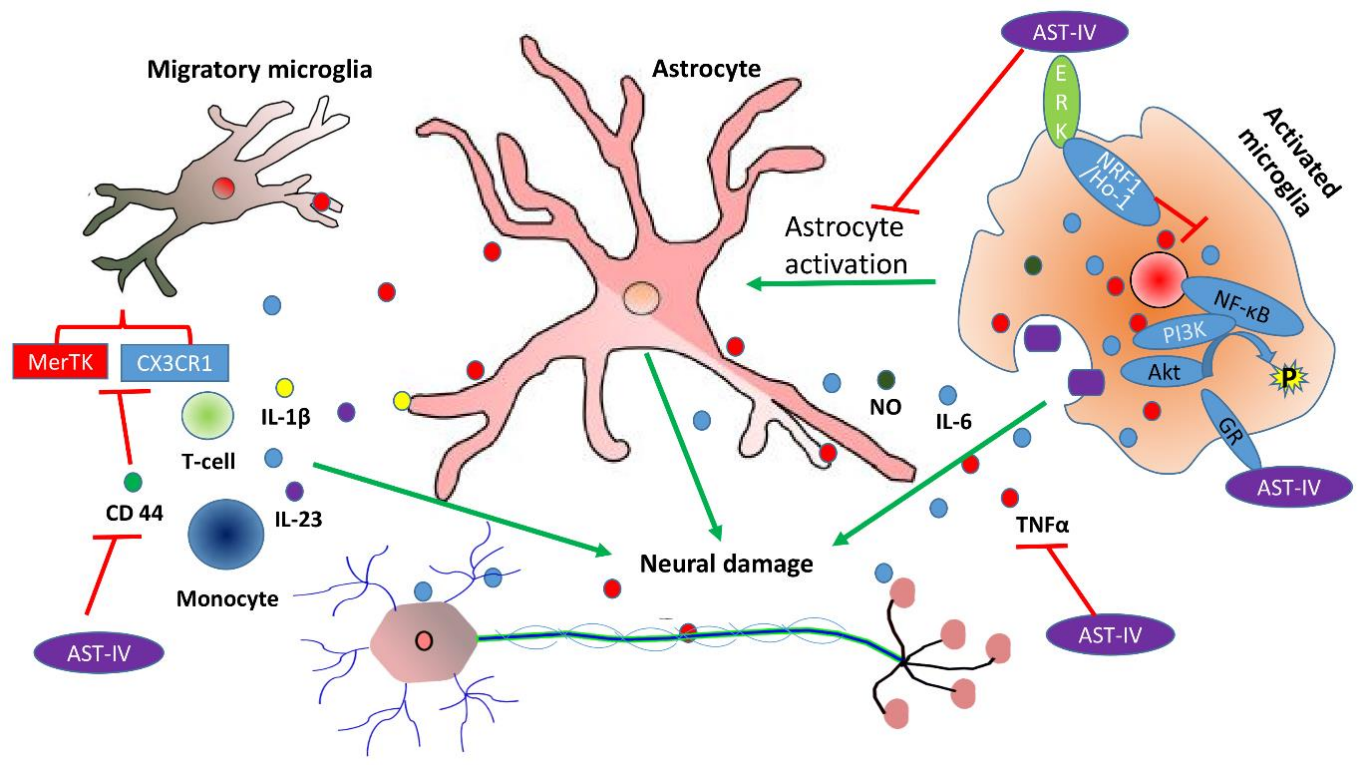
A schematic diagram illustrating the molecular mechanism underlying astragaloside IV-mediated protection of neurons against neuroinflammation. Astragaloside IV (denoted as AST-IV) reduces the migratory capability of microglia cells during inflammation to minimize neuronal loss. It also inhibits the activation of astrocytes, dephosphorylates Akt and PI3K proteins, activates the HRF1/Ho-1 cascade, and inhibits the generation of inflammasomes. Red lines represent inhibition; whereas green lines represent promotion.

Different inflammatory factors have previously been identified in activated microglial cells obtained from aged mice [44]. The levels of these factors (including CD44, CD14, CD86, CD11c, MHC-II, and programming ligand of death 1 marker (PD1) proteins change considerably during inflammation. These alterations collectively distressed the intracellular homeostasis mainly through downregulating the expression of *MerTK*, *Siglec-H* and *CX3CR1*, which induce changes and activate the microglia cells and positioning them as a hallmark of neural aging [44]. These changes further accompanied by an age-dependent increase in production of pro-inflammatory cytokines such as IL-6, TNF-α, and IL-1β which collectively promote the microglia cells senescence [45]. The activation of microglia both *in vivo* and *in vitro* has been reported to be significantly suppressed by astragaloside IV, mainly through promoting the activity of the glucocorticoid receptor-luciferase and enabling the translocation of the nuclear GR in microglial cells. Despite the relatively low affinity, astragaloside IV can bind to the GR and regulate the GR-mediated signalling pathways. The establishment of astragaloside IV-GR complex governs the dephosphorylation of various proteins (including Akt and PI3K), leading to a decrease in the production of pro-inflammatory mediators (Fig. 3) [46]. Astragaloside IV can also inhibit the activity of p16 protein and β-galactosidase to attenuate the premature senescence of astrocytes in the substantia nigra compacta region and to rehabilitate the dopaminergic neurons. Astragaloside IV mechanistically stimulates mitophagy that decreases the accumulation of damaged mitochondrial products and inhibits ROS generation to enhance astrocyte viability [47].

Apart from the mechanisms mentioned above, astragaloside IV enhances the extracellular receptors kinase (ERK) activation, triggering the NRF2/HO-1 cascade to lead to the anti-neuroinflammatory response in microglial cells [48]. It inhibits the expression of various genes (including *CASP3*, *COX-2*, and *Bax*) while up-regulating the expression of *Bcl-Xl*, *HO-1* and *Nrf2* to attenuate the neural inflammation and to promote the cell viability [49]. Furthermore, astragaloside IV can inhibit brain infiltration via modulating various intracellular mechanisms, such as the production of interferon-γ, deactivation of natural killer group 2D (NKG2D) receptors and histone deacetylases (HDAC), and by elevation of the level of acetylated p65 in astrocytes [50]. The bacterial endotoxin (lipopolysaccharide, LPS) is capable of triggering the activation of microglial cells [51]. Astragaloside IV attenuates the LPS-induced activation of microglial cells via down-regulating the pro-inflammatory (M1) mediators including nitric oxide (NO), interleukin 6 (IL-6), necrosis factor α (TNF-α), and interleukin (IL)-1β. It also increases the expression levels of diverse M2 mediators, including arginase 1 (*ARG1*), Toll-like receptors 4 (TLR4), and nuclear factor κB (NF-κB) in microglia [52]. Astragaloside IV alleviates LPS-induced ROS production *in vitro* and *in vivo* by inhibiting the expression of *NLRP3* and *Nrf2*. [53]. The phosphorylated-mitogen-activated protein kinase (p-MAPK) family is reported to be inhibited by astragaloside IV, which subsequently inhibits the inflammatory response in astrocytes [54]. Moreover, astragaloside IV strongly interacts with the immune system and protects astrocytes from damage through activation of the TLR3/NF-*κ*B pathway [55].

### 2.2. Enhancing genomic and mitochondrial integrity

Astragaloside IV plays multiple roles to maintain genomic integrity. It can ameliorate DNA damage and neurotoxicity by declining the level of glutaminase (GA), glutamine (Gln), glutamate (Glu) and glutamine synthetase (GS) while enhancing the amount of NO in the brain [56]. Astragaloside IV triggers the activation of the Nrf2/Keap1 cascade to inhibit inflammation and to hinders ROS production and apoptosis. This helps further maintain the genomic and morphological integrity of HK-2 cells [43]. Despite this, one study has found that astragaloside IV possesses anti-proliferative effects to inhibit the mitotic pathway and down-regulate DNA replication [57]. Moreover, astragaloside IV induces intrinsic/extrinsic apoptosis by triggering G_1_ arrest in HCC cells [58]. The exact mechanisms governing the effect of astragaloside IV on DNA replication and on the maintenance of genomic integrity are poorly elucidated at the moment and is an area that requires further investigation in future research.

**Figure 4.**
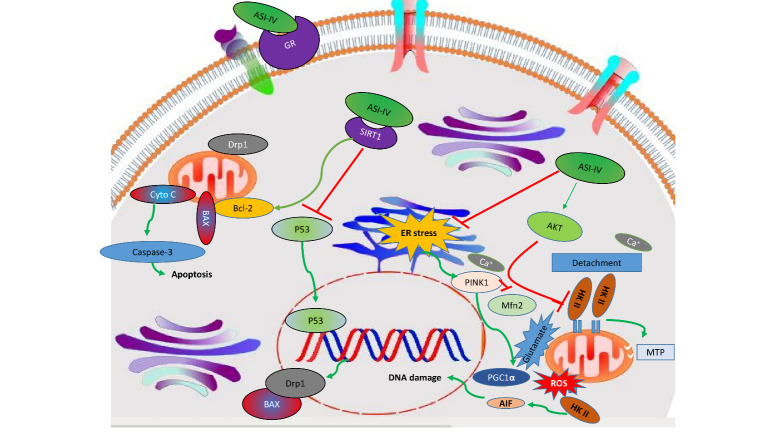
A schematic diagram depicting the protective effect of astragaloside IV on neuronal mitochondria. Red lines represent inhibition; whereas green lines represent promotion.

Apart from maintaining the genomic integrity, astragaloside IV modulates various cellular cascades to maintain the integrity of mitochondria. The permeability barrier of the inner mitochondrial membrane (IMM) sustains mitochondrial homeostasis and the binding of hexokinase-II (HK-II) to mitochondria [59]. Astragaloside IV enhances the survival of neurons by conserving HK-II in mitochondria and by increasing the expression of Akt protein. All these rescue the mitochondrial membrane potential and attenuate the production of apoptosis-inducing factors (AIF) [60]. Astragaloside IV can also sustain the mitochondrial membrane potential and the activity of the electron transport chain by down-regulating the expression of various genes (including *Drp1* and *BAX/BCL-2*) (Fig. 4) [61]. Mitochondria can crosstalk the endoplasmic reticulum (ER) via Ca^2+^ transport. This process is important to the maintenance of cellular homeostasis [62]. To combat the *ER* stress, astragaloside IV attenuates the expression of phosphor-protein kinase R-like ER kinase (p-PERK) and inositol-requiring ER-to-nucleus signal kinase 1 (IRE1), while promoting the phosphorylation of GSK-3*β* to protect the neurons [63]. Protein kinase A (PKA) triggers the activation of the cyclic AMP response element-binding protein (CREB) to shield the mitochondria from damage. The deprivation of glucose and oxygen in neurons impedes the activation of PKA and diminishes the phosphorylation of CREB to induce apoptosis in neurons. Astragaloside IV significantly enhances the PKA level and stimulates the phosphorylation of CREB to restore the mitochondrial activity [64]. It also attenuates various mitochondrial intrinsic cascades and increases the level of the FasL protein to ensure neural survival [65].

Amyloid-β (Aβ)-induced mitochondrial dysfunction plays a key role in the development of neurodegenerative disorders. The opening of the mitochondrial permeability transition pore (mPTP) is associated with Aβ-induced ROS production and neuronal cell senescence. Astragaloside IV is known to counteract the Aβ-induced changes in neurons by decreasing the superoxide level, inhibiting ROS generation, and by promoting the expression of B-cell lymphoma 2 (*Bcl-2*) [66]. Besides the opening of the mitochondrial permeability transition pore, Aβ triggers the phosphorylation c-Jun N-terminal kinase (JNK) through Toll-like receptor 4 (TLR4) protein [67]. Astragaloside IV shows a strong inhibitory effect on the phosphorylation of JNK in various organs [68], but the effect of JNK inhibition on neural cell surveillance has yet to be fully elucidated. Astragaloside IV also enhances the level of lamin B1 and promotes mitophagy to minimize mitochondrial damage [47]. The methionine sulfoxide reductase is an anti-oxidative enzyme that helps repair proteins damaged by oxidative stress. Through upregulation of sulfoxide reductase, astragaloside IV shows a protective effect on neurons against oxidative damage by recruiting the SIRT1/FOXO3 cascade [69]. All these enable astragaloside IV to serve as a potential therapeutic agent to overcome the metabolic disturbance led by neuronal aging.

### 2.3. Tackling calcium dysregulation and aberrant neurotransmission

The calcium ion (Ca^2+^) controls neuronal activities including long-term memory [70]. During aging, the capacity of neurons to regulate Ca^2+^ dynamics deteriorates, causing an increase in the Ca^2+^ influx from the ER through L-type voltage-dependent Ca^2+^ channels. This results in an abnormal rise in the cytoplasmic Ca^2+^ concentration, leading to changes in the cytoskeletal architecture, in gene expression and in the release of neurotransmitters [71-75]. Astragaloside IV not only decreases the magnitude of the current flow in voltage-gated K^+^ and Na^+^ channels but can also reduce the frequency of synchronized spontaneous oscillations of Ca^2+^ [76]. Moreover, activation of the mitochondrial Ca^2+^ uniporter (MCU) facilitates cytochrome C release, causes ATP depletion, and increases mitochondrial ROS generation. All these collectively lead to the death of neurons. By attenuating the excessive release of cytochrome C and by rescuing the mitochondrial Ca^2+^ overload, astragaloside IV decreases the aberrant MCU activation to maintain calcium homeostasis and neural viability [77]. Structural damage to neurons also influences neurotransmission in the brain. Heat, for example, reduces the level of acetylcholine. Astragaloside IV, however, can re-establish the acetylcholine level and has demonstrated the potential to treat central nervous system damage [78].

Astragaloside IV can improve the neural synaptic plasticity and cognitive function in mice, too, by suppressing the hippocampal transcription of *GAD65*, *EGR-1*, *TrkB* and *BDNF* [79]. This reverses neurobehavior deficit after ischemic stroke through BNDF/TrkB cascade [79]. Imbalanced release of neurotransmitters may lead to the occurrence of neuropsychiatric disorders (e.g., depressive-like behaviour and social interaction deficit). Astragaloside IV reverses neuropsychiatric symptoms and enhances cognitive functions by restoring the levels of various neurotransmitters, including monoamine oxidase (MAO-A), serotonin (5-HT), dopamine (DA), and tryptophan hydroxylase 2 (Tph2) [80]. Astragaloside IV can, therefore, be a candidate that warrants further exploitation as a therapeutic agent to stabilize the cellular level of calcium.

### 2.4. Stimulating stem cell renewal and neurogenesis

The pool of neural stem cells (NSCs) in the brain is exhausted with advanced age, leading to a gradual decrease in neurogenesis [81]. Telomere shortening is one of the possible causes of this [82] and may increase the risk of acquiring neurodegenerative diseases such as Alzheimer's disease [83]. Telomerase activity is mostly restricted to NSCs in the hippocampus dentate gyrus, subventricular zone, and few other parts of the brain [84]. Astragaloside VI can promote the self-renewal and proliferation of NSCs without altering their differentiation. In the *in vivo* context, it enhances the expression of p-MAPK and nestin and facilitates the activation of EGFR/MAPK pathway in the dentate gyrus zone, subventricular zone, and the cortex of the brain [85]. In addition, astragaloside IV shows positive effects on the differentiation and proliferation of engrafted NSCs. It stimulates the transition of NSCs into GFAP^+^ and tubulin III^+^ cells to increase the hippocampal density of tubulin III^+^ cells and hence toimprove cognitive abilities [86]. In addition, astragaloside IV potentially regulates *IL-17* expression. It also modulates the activity of the Akt/GSK-3 cascade to hinder neural apoptosis and to promote neurogenesis [87].

Furthermore, astragaloside IV can activate telomerase in a variety of cell types, particularly embryonic fibroblasts (MEFs, G3 *Terc*^+/-^) and hematopoietic progenitor cells. Astragaloside IV supplementation boosts *TERT* activation in the brain, liver, heart, lungs, and bone marrow to rescue telomere shorting in elderly mice [88, 89]. Astragaloside IV, in combination with cycloastragenol, promotes the activation of the Src/MEK/ERK pathway to enhance telomerase activity [90]. It upregulates the expression various genes such as pituitary homeobox 3 (*Ptx3*), dopamine transporter (*Dat*), Orphan nuclear hormone 1 (*Nurr1*), tyrosine hydroxylase (*Th*), Sonic hedgehog (*Shh*), to aid the proliferation and differentiation of dopaminergic neurons from NSCs [91]. Astragaloside IV also promotes neural regeneration by sustaining the elevated levels of growth-associated protein-43 (GAP-43) mRNA [92] and by activating the Wnt pathway [93]. Moreover, it reduces the build-up of advanced glycation end products and enhances glutathione peroxidase activity in nerves to promote regional demyelination and neurogenesis [39], while encouraging the regeneration of the neural wide gap and increasing the density of myelinated axons to hinder synaptic and neural loss and reduce cognitive impairment [94, 95].

## 3. Molecular mechanisms underlying the effects of astragaloside IV

### 3.1. Mammalian target of rapamycin (mTOR) pathway

mTOR kinase is an important regulator of various vital cellular events such as cell division, growth, and metabolism [96-100]. The correlation between mTOR and lifespan was initially demonstrated by Fabrizio and coworkers in invertebrates using gene-editing techniques [101]. mTOR can act as a both negative and positive regulator in the process of neural aging. For instance, autophagy and microglia M2 polarization maintain cell viability and homeostasis to protect neurons from apoptosis [102-104]. mTORC1 inhibits microglial M2 polarization and neuronal autophagy [105, 106], thereby promoting the mortality of neurons and escalating neural aging. On the other hand, mTOR helps sustain neurotransmission, synaptic plasticity, and neuronal viability to maintain neural development and function [107, 108]. Normally, mTOR expression is downregulated in an age-dependent manner [109], yet the mTOR pathway is hyperactive in both animal models and humans with Alzheimer's disease [110]. Treatment with rapamycin or rapalogs in mice with Alzheimer's disease reduces cognitive deterioration [111, 112], suggesting that inhibitors of mTOR can potentially heal age-related disorders though adverse effects (such as immune system suppression) are inevitable.

Astragaloside IV inhibits the mTORC1 signalling in microglial and neuronal cells. Administration of astragaloside IV to mice induces autophagy and promotes M2 polarization in neural cells, thereby inhibiting neuroinflammation [113]. Autophagy and interleukin 6 are key determents of neural aging [114, 115]. Lipopolysaccharides (LPS) inhibit autophagy and increase IL-6 production via the Akt/mTOR pathway in activated macrophages. Astragaloside IV suppresses the LPS-induced cellular autophagy and decreases the IL-6 level by triggering the activation of AMP-activated protein kinase (AMPK) to attenuate the mTOR cascade [116]. It also attenuates the apoptosis of neural cells by activating protein kinase B and phosphoinositide 3-kinase (PI3K), while markedly inhibiting the nuclear factor-κB (NF-κB) signalling cascade [117]. Here it is worth mentioning that there are limited or no data available yet to indicate the effect of astragaloside IV in promoting mTOR activity in the brain. In Caco-2 cell lines, astragaloside II has been found to improve L-arginine absorption and to activate the mTOR cascade to promote wound closure and cell proliferation. This suggests that astragaloside IV may have the capability of triggering the activation of mTOR. However, more investigations are needed to verify the association between astragaloside IV and its role in modulating the mTOR cascade during neural aging. Overall, mTOR plays a critical role in brain development (particularly in the formation of axons and dendrites, neural differentiation, and gliogenesis) and acts as a nutrition and growth factor sensor [118], it could be a potential target for the astragaloside IV-mediated treatment of neural aging.

### 3.2. Silent information regulator 1 (SIRT1) pathway

SIRT1 is a nicotinamide adenine dinucleotide (NAD^+^) dependent histone deacetylase. It is distributed all over the body and governs cellular metabolism by deacetylating histones and non-histone polypeptides in response to stress [119]. When genotoxic stress appears, SIRT1 migrates to DNA damage hotspots to lead to the upregulation of gene expression for DNA repair [120]. It also deacetylates the mitochondrial complexes I and III to increase electron transport capacity of mitochondria and to inhibit ROS generation [121].

Astragaloside IV modulates the activity of the SIRT1 pathway to regulate various cellular mechanisms. An intraperitoneal injection of astragaloside IV substantially increases SIRT1 expression, inactivates intracellular metalloproteinase-9, supresses the levels of pro-inflammatory cytokines (IL-1 and TNF-α), and hinders the nuclear translocation of NF-κB. All these lead to a decrease in the brain infarct volume, inhibit neuronal apoptosis, and reduces the rate of degradation of protected tight junctions [122]. Administration of astragaloside IV to mice promotes the expression of SIRT1 and activates the SIRT1/Mapt pathway, thereby inhibiting aberrant hyperphosphorylation and hyperacetylation of the microtubule-associated protein Tau, reducing cerebral infarction and rescuing neurological deficits [123]. In addition, astragaloside IV can up-regulate the level of glutathione (GSH) directly to maintain the structural and functional integrity of neurons [61].

Astragaloside IV recruits the SIRT1/FGF21/PPARα intracellular signalling pathway to overcome chronic inflammation, insulin resistance and aberrant glycolipid metabolism in the liver [124]. Interestingly, two of the important proteins of this pathway, namely FGF21 and SIRT1, have been reported to have a vital role in neurons. For example, FGF21 triggers the activation of PGC-1 through SIRT1, which promotes a rise in the nicotinamide phosphoribosyl transferase level and enhances mitochondrial respiratory capacity in the brain [125]. This suggests that the SIRT1/FGF21/PPARα pathway may have a similar function in the brain as reported in the liver to tackle metabolic abnormalities. Further research is needed to validate the association between the SIRT1/FGF21/PPARα pathway and neuronal survival so as to seek insights into the mechanisms underlying the onset and progression of neural diseases and aging at the molecular level.

### 3.3. Glucose metabolic pathway

Insulin plays an essential role in maintaining normal brain physiology [126, 127]. The disturbance in insulin/glucose metabolism promotes the production of advanced glycation products [128] and elevates the cytosolic glutamate level in neurons [129], resulting in neuro-inflammation and an increase in neural mortality. An excess of glutamate not only causes aberrant Ca^2+^ influx through NMDA receptors and induces neuronal injury [130], but can also trigger ROS production and promote neuronal cell senescence [131, 132]. Administration of astragaloside IV to glucose- and oxygen-deprived PC12 cells rescues mitochondria malfunction and ER stress, attenuates ROS generation, inhibits the activity of lactate dehydrogenase, and hinders apoptosis by activating of the p38 MAPK signalling cascade [133]. Astragaloside IV improves the levels of insulin, HbA1C, and glucose in blood and promotes the activity of glutathione peroxidase. Moreover, it inhibits the activity of aldose reductase in nerves to suppress the accumulation of advanced glycation end products in diabetic mice [39] and triggers the activation of the Raf/MEK/ERK pathway to attenuate the toxicity of PC12 cells [134].

More investigations are required to explore the role of different glucose metabolism-related signalling cascades in determining neuronal aging. For instance, while the sterol element regulatory binding protein-1c (SREBP-1c) cascade and the protein tyrosine phosphatase 1B (PTP1B) cascade can negatively affect glucose metabolism in hepatic cells [135], the functional role played by the SREBP-1c/PTP1B pathway in affecting glucose metabolism and hence the process of neuronal aging in the brain is not fully understood. This is one of the directions that warrant further studies. In addition, in muscle cells, astragaloside IV promotes the translocation of insulin-mediated glucose transporter 4 (GLUT4) to the plasma membrane and activates the IRS-l/PI 3-k/Akt signalling pathway to attenuate insulin resistance [136]. As IRS-l phosphorylation promotes glucose consumption, it is possible that this pathway may play a role in glucose consumption in neurons too. Yet, experimental verification is required to get an answer.

**Figure 5.**
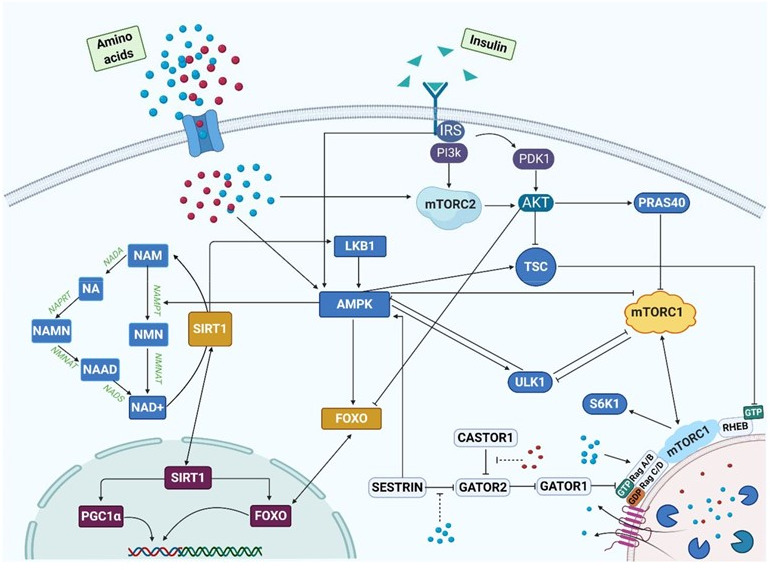
A schematic diagram illustrating astragaloside IV-mediated regulation of AMPK, SIRT1 and mTOR. The green lines represent promotion whereas the red lines represent inhibition. Blue circles represent leucine. Red circles represent arginine. Green triangles represent insulin. The diagram shows three distinct areas in the cell: the cytoplasm, lysosome, and the nucleus. Reproduced from ref. 145 with permission from Springer Nature.

### 3.4. AMPK pathway

AMPK is a cellular energy indicator, and crucial to cellular homeostasis. Upon activation, AMPK blocks the anabolic pathway to conserve cellular ATP [137, 138]. Till now, insulin-sensitizing compounds targeting the activity of AMPK have been discovered to treat hyperglycaemia [139]. Astragaloside IV also shows the ability to activate AMPK [140], leading to the down-regulation of the mTOR/Akt cascade to mitigate the effects of neural inflammation [116]. Moreover, AMPK enhances not only the viability of stem cells but also neurogenesis in the hippocampus [141]. For this, as an activator of AMPK, astragaloside IV may potentially enhance neurogenesis in specific parts of brain. Macrophage polarization shifts from the anti-inflammatory state to the pro-inflammatory one in an age-dependent manner, promoting inflammation and inducing apoptosis in neurons [142]. Astragaloside IV hinders this polarization process and inhibits the transcription of pro-inflammatory genes such as *CD206* to increase the proportion of M2 macrophages by activating the AMPK pathway [143]. Besides activating AMPK, astragaloside IV facilitates the transition of microglia/macrophages from M1 to M2 phenotypes to improve neuroplasticity and to restore the neurological function [144]. All the energy-sensing pathways are interconnected to manifest combined effects (Fig. 5) [145].

Similar to other energy-sensing pathways, the AMPK pathway necessitates further investigation to elucidate its role in neural aging. For example, functional deficiency of SREBP-1c has been reported to enhance lateral ventricle hypertrophy and to lead to impaired transmission of GABAnergic neurons [146]. In the brain, AMPK-dependent phosphorylation of SREBP-1c is known to reduce insulin resistance [140]. Astragaloside IV enhances the stability and phosphorylation of SREBP-1c in hepatic cells to attenuate the ER stress [147], suggesting that astragaloside IV has a similar effect in promoting the phosphorylation of SREBP-1c and the inhibition of SREBP-1 neurons. More studies are needed to explore the role played by astragaloside IV in modulating the activity of SREBP-1c/PTP1B in the brain and the subsequent effects on neural aging.

## 4. Effects of astragaloside IV on diseases associated with neuronal aging

AD is by far the most prevalent neurological disease associated with neuronal aging [148]. The hyper-phosphorylation of the Tau protein, the accumulation of Aβ and the formation of neurofibrillary tangles induce not only cognitive impairment but also the degradation of neurons to lead to the onset and progression of AD [149, 150]. Astragaloside IV combats various deleterious effects of AD by activating the PI3K/AKT and MAPK (or ERK) pathways. It also promotes the expression of synaptophysins and microtubule-associated protein 2 (MAP-2) to stimulate dendritic formation and to ameliorate cortical cell degeneration and memory loss in rats [151]. By activating the PPAR/BDNF signalling cascade, astragaloside IV can inhibit the Aβ-induced decrease in the BDNF level in the hippocampus and can mitigate AD-mediated neuronal anomalies [152]. Moreover, it acts as a preferential *PPAR* natural agonist in nerve cells and boosts *BACE1* expression to counteract the formation of neuritic plaques [153]. More recently, the association between microtubule associated protein tau (MAPT) and AD has been explored [154]. The hyper-phosphorylation of MAPT results in the formation of neurofibrillary tangles and promotes neural senescence. The acetylation of MAPT can reverse these pathogenic effects by decreasing neurofibrillary tangle formation [155]. Astragaloside IV up-regulates the activity of *SIRT1* to reduce aberrant hyper-phosphorylation of MAPT and to modulate the downstream events of MAPT to halt the production of neurofibrillary tangles in rats [123]. Finally, by attenuating intracellular ROS generation, astragaloside IV can inhibit mPTP opening and can reduce the mitochondrial superoxide level in SK-N-SH cells to increase the neuronal viability [66].

Apart from the onset and progression of AD, those of Parkinson's disease (PD) (which is characterized by the atrophy of dopaminergic neurons in the substantia nigra pars compacta and by the reduction in the dopamine level in the striatum [156]) can be modulated by using astragaloside IV. The possible use of astragaloside IV to tackle behavioural deficits caused by 1-methyl-4-phenyl-1,2,3,6-tetrahydropyridine (MPTP)-induced parkin-sonism has been investigated by Xia and coworkers [157]. Astragaloside IV has been found to substantially combat the behavioural deficits and to restore cell viability by enhancing the expression of caspase 3 protein, by increasing the level of p-JNK, and by boosting the Bax/Bcl-2 ratio. Astragaloside IV also promotes the lamin B1 level and reduces the level of pro-inflammatory proteins, thereby protecting dopamine neurons in the substantia nigra compact and ameliorating behavioural impairments in mice [47]. By activating the NFκB/NLRP3 signalling pathway, astragaloside IV triggers antioxidant and anti-inflammatory effects against MPTP-induced dopamine neurons degradation in mice [53]. It also protects nerve cells by reducing the level of C/EBP-homologous protein (CHOP) and by inhibiting lincRNA-p21 expression to ameliorate the ER stress [158].

## 5. Challenges and future prospects

Toxicity is one of the issues to be considered before astragaloside IV is used practically for treatment development. This need is partially demonstrated by a recent study [159], in which rats have received daily intravenous administration of astragaloside IV from day 6 after gestation to day 15. An increase in the proportion of visible dead foetuses has been observed in the treatment group. A similar observation has been made in rabbits which have been injected intravenously with astragaloside IV from day 6 after gestation to day 18 [159]. More recently, Wan and co-workers have also reported that when Sprague-Dawley rats have been fed with astragaloside IV at a dose of 1.0 mg/kg for 28 days, fur development, eye opening, and cliff parry reflex of their pups are delayed [160]. Astragaloside IV should, therefore, be administered cautiously to children and perinatal women. Moreover, the altered expression of *Notch1* is associated with the morbidity of AD [161]. A low dose of astragaloside IV up-regulates the expression of *Notch1*; whereas a high dose of it not only does the other way round [86] but also impedes nerve regeneration [94]. In fact, proper evaluation of the toxicity of astragaloside IV is challenging. For instance, astragaloside IV triggers the immune system and may increase the risk of getting autoimmune diseases in patients [162]. It may also induce symptoms (e.g., an increase in the nerve conduction velocity and the mechanical withdrawal threshold) of neurotoxicity in rats [38]. Administration of astragaloside IV has also been found to promote the expression of telomerase via modulation of the MAPK, JAK/STAT, and CREB cascades [163], and to promote the angiogenesis by activating the AKT/GSK-3β/β catenin signalling pathway [164]. More research is needed to explore the possible effect of astragaloside IV on the onset and metastasis of cancer because cancer is associated with abnormal telomerase activity and angiogenesis. All these suggest that administration of astragaloside IV at an improper dose may adversely affect the treatment outcome.

Bioavailability is another factor to be considered when astragaloside IV is used as a therapeutic agent. The oral bioavailability of astragaloside IV is around 7% in dogs and less than 5% in rats [34]. In Caco-2 cells, antagonists of P-glycoprotein have shown no effect on the cellular uptake of astragaloside IV, suggesting that the low bioavailability of astragaloside IV is not caused by this efflux protein [165]. Lack of target specificity is another factor that may reduce the therapeutic effect of astragaloside IV upon administration to a living body. The development of nanotechnologies is one possible approach to enhance the bioavailability and biocompatibility of bioactive compounds [166-171]. This is demonstrated by the case of Fe_3_O_4_-astragaloside IV nanoparticles, which show enhanced stability, high aqueous solubility and low toxicity for treatment of anaemia [172]. More studies on the design and engineering of astragaloside IV-loaded nanoparticles can bring a vista of new opportunities for the development of new interventions against neural aging.

Finally, herbal medicines have been widely known as a key source of bioactive agents to treat neurological disorders and malignancies [173]. They may give a synergistic effect when used in combination with chemical drugs. For instance, comparing with the rat models treated with either astragaloside IV or ligustrazine, those treated with both agents concomitantly show a more significant size reduction in the cerebral lesion area. This is because the combined use of both agents can more strongly modulate the activity of intracellular regulatory factors of T cells to ameliorate neuroinflammation [174]. Using astragaloside IV and polyurethane concurrently also dramatically increases the levels of neuronal regeneration indicators and promotes the proliferation of Schwann cells in mice [175]. Along with the observation that the combined use of atorvastatin and astragaloside IV can more effectively reduce the inflammatory response in mice than either of the two agents does [176], integrating astragaloside IV into the regimen of chemical drugs is a possible strategy to enhance the therapeutic efficiency when treatment is developed to tackle neuronal aging. Nevertheless, possible interactions of co-delivered agents is a complicated problem when multi-drug therapy is applied [177-181]. Efforts should be put to evaluate the safety and efficiency of astragaloside IV-containing multi-drug regimens on a case-by-case basis.

## 6. Conclusion

Astragaloside IV has a wide spectrum of pharmacological activities on the central nervous system [182]. It can ameliorate a range of neurological aging hallmarks including mitochondrial dysfunction, alterations in energy-sensing pathways, abnormal release of Ca^+^ and neurotransmitters, and a decline in cognitive function. Astragaloside IV shows the capacity of suppressing microglial activation, combating ROS generation and inflammation, and enhancing the level of neurotrophins. The safety, bioavailability and target specificity are some of the factors to be considered when astragaloside IV-based regimens are adopted to tackle neuronal aging. Nevertheless, with the advances in nanotechnologies [167, 168, 183], some of the problems (including the low bioavailability and lack of target specificity) associated with the therapeutic use of astragaloside IV should be able to be addressed. Last but not least, till now most of the studies on the biological activity of astragaloside IV are performed *in vitro* or *in vivo*, studies examining the therapeutic effect of the agent in the clinical context is lacking. More efforts are needed in the future to not only validate the clinical potential of astragaloside IV but also to extend the knowledge of the toxicity and pharmacokinetics of that agent in a human body.

## References

[1] Mattson MP, Magnus T (2006). Ageing and neuronal vulnerability. Nat Rev Neurosci, 7(4):278-294. 16552414 10.1038/nrn1886 PMC3710114

[2] Chen C, Turnbull DM, Reeve AK (2019). Mitochondrial dysfunction in Parkinson’s disease—cause or consequence? Biology (Basel), 8(2):38. 10.3390/biology8020038 PMC6627981 31083583

[3] Rivera A, Vanzuli I, Julio Rodríguez Arellano J, Butt A (2016). Decreased regenerative capacity of oligodendrocyte progenitor cells (NG2-glia) in the ageing brain: a vicious cycle of synaptic dysfunction, myelin loss and neuronal disruption? Curr Alzheimer Res, 13(4):413-418. 26567743 10.2174/1567205013666151116125518

[4] Foster TC, Kumar A (2002). Calcium dysregulation in the aging brain. The Neuroscientist, 8(4):297-301. 12194497 10.1177/107385840200800404

[5] De Lucia C, Murphy T, Steves CJ, Dobson RJ, Proitsi P, Thuret S (2020). Lifestyle mediates the role of nutrient-sensing pathways in cognitive aging: cellular and epidemiological evidence. Commun Biol, 3(1):1-17. 32242137 10.1038/s42003-020-0844-1 PMC7118127

[6] Bishop NA, Lu T, Yankner BA (2010). Neural mechanisms of ageing and cognitive decline. Nature, 464(7288):529-535. 20336135 10.1038/nature08983 PMC2927852

[7] Alexander GE, Ryan L, Bowers D, Foster TC, Bizon JL, Geldmacher DS, et al. (2012). Characterizing cognitive aging in humans with links to animal models. Front Aging Neurosci, 4:21. 22988439 10.3389/fnagi.2012.00021 PMC3439638

[8] Dykiert D, Der G, Starr JM, Deary IJ (2012). Age differences in intra-individual variability in simple and choice reaction time: systematic review and meta-analysis. PLoS One, 7(10):e45759. 23071524 10.1371/journal.pone.0045759 PMC3469552

[9] Levin O, Fujiyama H, Boisgontier MP, Swinnen SP, Summers JJ (2014). Aging and motor inhibition: a converging perspective provided by brain stimulation and imaging approaches. Neurosci Biobehav Rev, 43:100-117. 24726575 10.1016/j.neubiorev.2014.04.001

[10] Peters R (2006). Ageing and the brain. Postgrad Med J, 82(964):84-88. 16461469 10.1136/pgmj.2005.036665 PMC2596698

[11] Dawson TM, Golde TE, Lagier-Tourenne C (2018). Animal models of neurodegenerative diseases. Nat Neurosci, 21(10):1370-1379. 30250265 10.1038/s41593-018-0236-8 PMC6615039

[12] Campos PB, Paulsen BS, Rehen SK (2014). Accelerating neuronal aging in in vitro model brain disorders: a focus on reactive oxygen species. Front Aging Neurosci, 6:292. 25386139 10.3389/fnagi.2014.00292 PMC4209886

[13] Son TG, Camandola S, Arumugam TV, Cutler RG, Telljohann RS, Mughal MR, et al. (2010). Plumbagin, a novel Nrf2/ARE activator, protects against cerebral ischemia. J Neurochem, 112(5):1316-1326. 20028456 10.1111/j.1471-4159.2009.06552.x PMC2819586

[14] Yang C, Zhang X, Fan H, Liu Y (2009). Curcumin upregulates transcription factor Nrf2, HO-1 expression and protects rat brains against focal ischemia. Brain Res, 1282:133-141. 19445907 10.1016/j.brainres.2009.05.009

[15] Srividhya R, Zarkovic K, Stroser M, Waeg G, Zarkovic N, Kalaiselvi P (2009). Mitochondrial alterations in aging rat brain: effective role of (-)-epigallo catechin gallate. Int J Dev Neurosci, 27(3):223-231. 19429387 10.1016/j.ijdevneu.2009.01.003

[16] Unno K (2016). Prevention of brain aging by green tea components: role of catechins and theanine. J Phys Fit Sports Med, 5(2):117-122.

[17] Ide K, Matsuoka N, Yamada H, Furushima D, Kawakami K (2018). Effects of tea catechins on Alzheimer's disease: recent updates and perspectives. Molecules, 23(9):2357. 10.3390/molecules23092357 PMC6225145 30223480

[18] Eyolfson E, Malik H, Mychasiuk R (2020). Sexually dimorphic behavioral and genetic outcomes associated with administration of TA65 (a telomerase activator) following repetitive traumatic brain injury: a pilot study. Front Neurol, 11:98. 32132968 10.3389/fneur.2020.00098 PMC7040363

[19] Liu Y, Gao J, Peng M, Meng H, Ma H, Cai P, et al. (2018). A review on central nervous system effects of gastrodin. Front Pharmacol, 9:24-24. 29456504 10.3389/fphar.2018.00024 PMC5801292

[20] Sarveazad A, Babahajian A, Yari A, Goudarzi F, Soleimani M, Nourani M (2017). Neuroprotective role of trolox in hippocampus after ischemia reperfusion injury in mouse. Int J Vitam Nutr Res, 1(1):1-7. 10.1024/0300-9831/a000293 28485696

[21] Saito S, Tanaka M, Satoh-Asahara N, Carare RO, Ihara M (2021). Taxifolin: a potential therapeutic agent for cerebral amyloid angiopathy. Front Pharmacol, 12:643357-643357. 33643053 10.3389/fphar.2021.643357 PMC7907591

[22] Kouhestani S, Jafari A, Babaei P (2018). Kaempferol attenuates cognitive deficit via regulating oxidative stress and neuroinflammation in an ovariectomized rat model of sporadic dementia. Neural Regen Res, 13(10):1827-1832. 30136699 10.4103/1673-5374.238714 PMC6128063

[23] Wang K-J, Zhang W-Q, Liu J-J, Cui Y, Cui J-Z (2020). Piceatannol protects against cerebral ischemia/reperfusion-induced apoptosis and oxidative stress via the Sirt1/FoxO1 signaling pathway. Mol Med Rep, 22(6):5399-5411. 33173979 10.3892/mmr.2020.11618 PMC7647030

[24] Grewal R, Reutzel M, Dilberger B, Hein H, Zotzel J, Marx S, et al. (2020). Purified oleocanthal and ligstroside protect against mitochondrial dysfunction in models of early Alzheimer's disease and brain ageing. Exp Neurol, 328:113248. 32084452 10.1016/j.expneurol.2020.113248

[25] Nakhate KT, Bharne AP, Verma VS, Aru DN, Kokare DM (2018). Plumbagin ameliorates memory dysfunction in streptozotocin induced Alzheimer’s disease via activation of Nrf2/ARE pathway and inhibition of β-secretase. Biomed Pharmacother, 101:379-390. 29501041 10.1016/j.biopha.2018.02.052

[26] Song J, Li N, Xia Y, Gao Z, Zou S-F, Kong L, et al. (2016). Arctigenin treatment protects against brain damage through an anti-inflammatory and anti-apoptotic mechanism after needle insertion. Front Pharmacol, 7:182-182. 27445818 10.3389/fphar.2016.00182 PMC4916177

[27] Bloemendaal M, Froböse MI, Wegman J, Zandbelt BB, van de Rest O, Cools R, et al. (2018). Neuro-cognitive effects of acute tyrosine administration on reactive and proactive response inhibition in healthy older adults. eNeuro, 5(2):ENEURO.0035-0017.2018. 10.1523/ENEURO.0035-17.2018 PMC6084775 30094335

[28] Wu S, Yue Y, Peng A, Zhang L, Xiang J, Cao X, et al. (2016). Myricetin ameliorates brain injury and neurological deficits via Nrf2 activation after experimental stroke in middle-aged rats. Food Funct, 7(6):2624-2634. 27171848 10.1039/c6fo00419a

[29] Hussain G, Huang J, Rasul A, Anwar H, Imran A, Maqbool J, et al. (2019). Putative roles of plant-derived tannins in neurodegenerative and neuropsychiatry disorders: an updated review. Molecules, 24(12):2213. 10.3390/molecules24122213 PMC6630756 31200495

[30] Li H, Chen FJ, Yang WL, Qiao HZ, Zhang SJ (2021). Quercetin improves cognitive disorder in aging mice by inhibiting NLRP3 inflammasome activation. Food Funct, 12(2):717-725. 33338087 10.1039/d0fo01900c

[31] Zhu Y, Wang K, Ma Z, Liu D, Yang Y, Sun M, et al. (2019). SIRT1 activation by butein attenuates sepsis-induced brain injury in mice subjected to cecal ligation and puncture via alleviating inflammatory and oxidative stress. Toxicol Appl Pharmacol, 363:34-46. 30336174 10.1016/j.taap.2018.10.013

[32] Ren S, Zhang H, Mu Y, Sun M, Liu P (2013). Pharmacological effects of Astragaloside IV: a literature review. J Tradit Chin Med, 33(3):413-416. 24024343 10.1016/s0254-6272(13)60189-2

[33] Costa IM, Lima FOV, Fernandes LCB, Norrara B, Neta FI, Alves RD, et al. (2019). Astragaloside IV supplementation promotes a neuroprotective effect in experimental models of neurological disorders: a systematic review. Curr Neuropharmacol, 17(7):648-665. 30207235 10.2174/1570159X16666180911123341 PMC6712289

[34] Zhang Q, Zhu L-L, Chen G-G, Du Y (2007). Pharmacokinetics of astragaloside iv in beagle dogs. Eur J Drug Metab Pharmacokinet, 32(2):75-79. 17702194 10.1007/BF03190995

[35] Huang C, Wang G, Wu X, Li H, Xie H, Lv H, et al. (2006). Absorption enhancement study of astragaloside IV based on its transport mechanism in caco-2 cells. Eur J Drug Metab Pharmacokinet, 31(1):5-10. 16715776 10.1007/BF03190635

[36] Huang X-P, Qiu Y-Y, Wang B, Ding H, Tang Y-H, Zeng R, et al. (2014). Effects of Astragaloside IV combined with the active components of Panax notoginseng on oxidative stress injury and nuclear factor-erythroid 2-related factor 2/heme oxygenase-1 signaling pathway after cerebral ischemia-reperfusion in mice. Pharmacogn Mag, 10(40):402-409. 25422538 10.4103/0973-1296.141765 PMC4239715

[37] Shao A, Guo S, Tu S, Ammar A-b, Tang J, Hong Y, et al. (2014). Astragaloside IV alleviates early brain injury following experimental subarachnoid hemorrhage in rats. Int J Med Sci, 11(10):1073-1081. 25136262 10.7150/ijms.9282 PMC4135229

[38] Xu J, Guan Z, Wang X, Sun D, Li Y, Pei B, et al. (2021). Network pharmacology and experimental evidence identify the mechanism of astragaloside IV in oxaliplatin neurotoxicity. Drug Des Devel Ther, 15:99-110. 10.2147/DDDT.S262818 PMC7811377 33469263

[39] Yu J, Zhang Y, Sun S, Shen J, Qiu J, Yin X, et al. (2006). Inhibitory effects of astragaloside IV on diabetic peripheral neuropathy in rats. Can J Physiol Pharmacol, 84(6):579-587. 16900242 10.1139/y06-015

[40] Kim S, Kang IH, Nam JB, Cho Y, Chung DY, Kim SH, et al. (2015). Ameliorating the effect of astragaloside IV on learning and memory deficit after chronic cerebral hypoperfusion in rats. Molecules, 20(2):1904-1921. 25625683 10.3390/molecules20021904 PMC6272750

[41] Liu X, Zhang J, Wang S, Qiu J, Yu C (2017). Astragaloside IV attenuates the H2O2-induced apoptosis of neuronal cells by inhibiting α-synuclein expression via the p38 MAPK pathway. Int J Mol Med, 40(6):1772-1780. 29039448 10.3892/ijmm.2017.3157 PMC5716437

[42] Hao M, Liu Y, Chen P, Jiang H, Kuang H-Y (2018). Astragaloside IV protects RGC-5 cells against oxidative stress. Neural Regen Res, 13(6):1081-1086. 29926836 10.4103/1673-5374.233452 PMC6022471

[43] Han J, Guo D, Sun XY, Wang JM, Ouyang JM, Gui BS (2019). Repair effects of astragalus polysaccharides with different molecular weights on oxidatively damaged HK-2 cells. Sci Rep, 9(1):1-15. 31285477 10.1038/s41598-019-46264-y PMC6614371

[44] Mrdjen D, Pavlovic A, Hartmann FJ, Schreiner B, Utz SG, Leung BP, et al. (2018). High-dimensional single-cell mapping of central nervous system immune cells reveals distinct myeloid subsets in health, aging, and disease. Immunity, 48(2):380-395. 29426702 10.1016/j.immuni.2018.01.011

[45] Ritzel RM, Crapser J, Patel AR, Verma R, Grenier JM, Chauhan A, et al. (2016). Age-associated resident memory CD8 T cells in the central nervous system are primed to potentiate inflammation after ischemic brain injury. J Immunol, 196(8):3318-3330. 26962232 10.4049/jimmunol.1502021 PMC4868658

[46] Liu H-S, Shi H-L, Huang F, Peterson KE, Wu H, Lan Y-Y, et al. (2016). Astragaloside IV inhibits microglia activation via glucocorticoid receptor mediated signaling pathway. Sci Rep, 6(1):1-14. 26750705 10.1038/srep19137 PMC4707476

[47] Xia M-L, Xie X-H, Ding J-H, Du R-H, Hu G (2020). Astragaloside IV inhibits astrocyte senescence: implication in Parkinson’s disease. J Neuroinflammation, 17:1-13. 32252767 10.1186/s12974-020-01791-8 PMC7137443

[48] Li C, Yang F, Liu F, Li D, Yang T (2018). NRF2/HO-1 activation via ERK pathway involved in the anti-neuroinflammatory effect of Astragaloside IV in LPS induced microglial cells. Neurosci Lett, 666:104-110. 29273400 10.1016/j.neulet.2017.12.039

[49] Chen X, Cheng C, Zuo X, Huang W (2020). Astragalin alleviates cerebral ischemia-reperfusion injury by improving anti-oxidant and anti-inflammatory activities and inhibiting apoptosis pathway in rats. BMC Complement Med Ther, 20:1-11. 32316944 10.1186/s12906-020-02902-x PMC7171805

[50] Dou B, Li S, Wei L, Wang L, Zhu S, Wang Z, et al. (2021). Astragaloside IV suppresses post-ischemic natural killer cell infiltration and activation in the brain: involvement of histone deacetylase inhibition. Front Med, 15(1):79-90. 33369712 10.1007/s11684-020-0783-8

[51] Lively S, Schlichter LC (2018). Microglia responses to pro-inflammatory stimuli (LPS, IFNγ+TNFα) and reprogramming by resolving cytokines (IL-4, IL-10). Front Cell Neurosci, 12:215-215. 30087595 10.3389/fncel.2018.00215 PMC6066613

[52] Yu J, Guo M, Li Y, Zhang H, Chai Z, Wang Q, et al. (2019). Astragaloside IV protects neurons from microglia-mediated cell damage through promoting microglia polarization. 57(2):170-181. 10.5114/fn.2019.86299 31556576

[53] Yang C, Mo Y, Xu E, Wen H, Wei R, Li S, et al. (2019). Astragaloside IV ameliorates motor deficits and dopaminergic neuron degeneration via inhibiting neuroinflammation and oxidative stress in a Parkinson's disease mouse model. Int Immunopharmacol, 75:105651. 31401385 10.1016/j.intimp.2019.05.036

[54] Zhu X, Chen Y, Du Y, Wan Q, Xu Y, Wu J (2018). Astragaloside IV attenuates penicillin-induced epilepsy via inhibiting activation of the MAPK signaling pathway. Mol Med Rep, 17(1):643-647. 29115438 10.3892/mmr.2017.7896

[55] Shi L, Yin F, Xin X, Mao S, Hu P, Zhao C, et al. (2014). Astragalus polysaccharide protects astrocytes from being infected by HSV-1 through TLR3/NF-B signaling pathway. Evid Based Complement Alternat Med, 2014:285356. 10.1155/2014/285356 PMC4098889 25057274

[56] Essawy AE, Abd Elkader H-TAE, Khamiss OA, Eweda SM, Abdou HM (2021). Therapeutic effects of astragaloside IV and Astragalus spinosus saponins against bisphenol A-induced neurotoxicity and DNA damage in rats. PeerJ, 9:e11930. 34434659 10.7717/peerj.11930 PMC8359804

[57] Lee G-S, Jeong H-Y, Yang H-G, Seo Y-R, Jung E-G, Lee Y-S, et al. (2021). Astragaloside IV suppresses hepatic proliferation in regenerating rat liver after 70% partial hepatectomy via down-regulation of cell cycle pathway and DNA replication. Molecules, 26(10):2895. 34068164 10.3390/molecules26102895 PMC8152973

[58] Su CM, Wang HC, Hsu FT, Lu CH, Lai CK, Chung JG, et al. (2020). Astragaloside IV induces apoptosis, G1-phase arrest and inhibits anti-apoptotic signaling in hepatocellular carcinoma. In Vivo, 34(2):631-638. 32111763 10.21873/invivo.11817 PMC7157888

[59] Nederlof R, Eerbeek O, Hollmann MW, Southworth R, Zuurbier CJ (2014). Targeting hexokinase II to mitochondria to modulate energy metabolism and reduce ischaemia-reperfusion injury in heart. Br J Pharmacol, 171(8):2067-2079. 24032601 10.1111/bph.12363 PMC3976622

[60] Li Y, Yang Y, Zhao Y, Zhang J, Liu B, Jiao S, et al. (2019). Astragaloside IV reduces neuronal apoptosis and parthanatos in ischemic injury by preserving mitochondrial hexokinase-II. Free Radic Biol Med, 131:251-263. 30502455 10.1016/j.freeradbiomed.2018.11.033

[61] Ben Y, Hao J, Zhang Z, Xiong Y, Zhang C, Chang Y, et al. (2021). Astragaloside IV inhibits mitochondrial-dependent apoptosis of the dorsal root ganglion in diabetic peripheral neuropathy rats through modulation of the SIRT1/p53 signaling pathway. Diabetes Metab Syndr Obes, 14:1647. 33883914 10.2147/DMSO.S301068 PMC8055373

[62] Malhotra JD, Kaufman RJ (2011). ER stress and its functional link to mitochondria: role in cell survival and death. Cold Spring Harb Perspect Biol, 3(9):a004424-a004424. 21813400 10.1101/cshperspect.a004424 PMC3181038

[63] Fu Y, Cai J, Xi M, He Y, Zhao Y, Zheng Y, et al. (2020). Neuroprotection effect of astragaloside IV from 2-DG-induced endoplasmic reticulum stress. Oxid Med Cell Longev, 2020:9782062. 33488941 10.1155/2020/9782062 PMC7790552

[64] Xue B, Huang J, Ma B, Yang B, Chang D, Liu J (2019). Astragaloside IV protects primary cerebral cortical neurons from oxygen and glucose deprivation/reoxygenation by activating the PKA/CREB pathway. Neuroscience, 404:326-337. 30708047 10.1016/j.neuroscience.2019.01.040

[65] Yin F, Zhou H, Fang Y, Li C, He Y, Yu L, et al. (2020). Astragaloside IV alleviates ischemia reperfusion-induced apoptosis by inhibiting the activation of key factors in death receptor pathway and mitochondrial pathway. J Ethnopharmacol, 248:112319. 31639488 10.1016/j.jep.2019.112319

[66] Sun Q, Jia N, Wang W, Jin H, Xu J, Hu H (2014). Protective effects of astragaloside IV against amyloid beta1-42 neurotoxicity by inhibiting the mitochondrial permeability transition pore opening. PLoS One, 9(6):e98866. 24905226 10.1371/journal.pone.0098866 PMC4048237

[67] Nagai H, Noguchi T, Takeda K, Ichijo H (2007). Pathophysiological roles of ASK1-MAP kinase signaling pathways. BMB Rep, 40(1):1-6. 10.5483/bmbrep.2007.40.1.001 17244475

[68] Xu W, Shao X, Tian L, Gu L, Zhang M, Wang Q, et al. (2014). Astragaloside IV ameliorates renal fibrosis via the inhibition of mitogen-activated protein kinases and antiapoptosis in vivo and in vitro. J Pharmacol Exp Ther, 350(3):552-562. 24951279 10.1124/jpet.114.214205

[69] Liu Y, Chong L, Li X, Tang P, Liu P, Hou C, et al. (2017). Astragaloside IV rescues MPP+-induced mitochondrial dysfunction through upregulation of methionine sulfoxide reductase A. Exp Ther Med, 14(3):2650-2656. 28962208 10.3892/etm.2017.4834 PMC5609276

[70] Cohen SM, Li B, Tsien RW, Ma H (2015). Evolutionary and functional perspectives on signaling from neuronal surface to nucleus. Biochem Biophys Res Commun, 460(1):88-99. 25998737 10.1016/j.bbrc.2015.02.146 PMC4701207

[71] Thibault O, Hadley R, Landfield PW (2001). Elevated postsynaptic [Ca^2^+]_i_ and L-type calcium channel activity in aged hippocampal neurons: relationship to impaired synaptic plasticity. J Neurosci, 21(24):9744-9756. 11739583 10.1523/JNEUROSCI.21-24-09744.2001 PMC6763040

[72] Toescu EC, Verkhratsky A, Landfield PW (2004). Ca^2^+ regulation and gene expression in normal brain aging. Trends Neurosci, 27(10):614-620. 15374673 10.1016/j.tins.2004.07.010

[73] Gant JC, Sama MM, Landfield PW, Thibault O (2006). Early and simultaneous emergence of multiple hippocampal biomarkers of aging is mediated by Ca^2^+-induced Ca^2^+ release. J Neurosci, 26(13):3482-3490. 16571755 10.1523/JNEUROSCI.4171-05.2006 PMC6673869

[74] Porte Y, Buhot MC, Mons N (2008). Alteration of CREB phosphorylation and spatial memory deficits in aged 129T2/Sv mice. Neurobiol Aging, 29(10):1533-1546. 17478013 10.1016/j.neurobiolaging.2007.03.023

[75] Südhof TC (2012). Calcium control of neurotransmitter release. Cold Spring Harb Perspect Biol, 4(1):a011353-a011353. 22068972 10.1101/cshperspect.a011353 PMC3249630

[76] Zhu SQ, Qi L, Rui YF, Li RX, He XP, Xie ZP (2008). Astragaloside IV inhibits spontaneous synaptic transmission and synchronized Ca^2^+ oscillations on hippocampal neurons. Acta Pharmacol Sin, 29(1):57-64. 18158866 10.1111/j.1745-7254.2008.00712.x

[77] Dong Z, Zhang C, Chen Y, Chen Y, Yuan Z, Peng Y, et al. (2017). Astragaloside-IV protects against heat-induced apoptosis by inhibiting excessive activation of mitochondrial Ca^2^+ uniporter. Cell Physiol Biochem, 42(2):480-494. 28578342 10.1159/000477595

[78] Zhao L, Sun Y, Yu C, Chen J, Xu X, Zhang X, et al. (2020). Astragaloside protects rat brain from microwave-induced functional injuries via restoring acetylcholine and normalizing electroencephalogram. Environ Sci Pollut Res Int, 27(32):40787-40794. 32677014 10.1007/s11356-020-07915-0

[79] Ni GX, Liang C, Wang J, Duan CQ, Wang P, Wang YL (2020). Astragaloside IV improves neurobehavior and promotes hippocampal neurogenesis in MCAO rats though BDNF-TrkB signaling pathway. Biomed Pharmacother, 130:110353. 32682983 10.1016/j.biopha.2020.110353

[80] Abd Elkader HAE, Abdou HM, Khamiss OA, Essawy AE (2021). Anti-anxiety and antidepressant-like effects of astragaloside IV and saponins extracted from Astragalus spinosus against the bisphenol A-induced motor and cognitive impairments in a postnatal rat model of schizophrenia. Environ Sci Pollut Res Int. 28(26):35171-35187. 33666843 10.1007/s11356-021-12927-5

[81] Encinas JM, Michurina TV, Peunova N, Park J-H, Tordo J, Peterson DA, et al. (2011). Division-coupled astrocytic differentiation and age-related depletion of neural stem cells in the adult hippocampus. Cell stem cell, 8(5):566-579. 21549330 10.1016/j.stem.2011.03.010 PMC3286186

[82] Ferrón SR, Marqués-Torrejón MÁ, Mira H, Flores I, Taylor K, Blasco MA, et al. (2009). Telomere shortening in neural stem cells disrupts neuronal differentiation and neuritogenesis. J Neurosci, 29(46):14394-14407. 19923274 10.1523/JNEUROSCI.3836-09.2009 PMC6665809

[83] Forero DA, González-Giraldo Y, López-Quintero C, Castro-Vega LJ, Barreto GE, Perry G (2016). Meta-analysis of telomere length in Alzheimer’s disease. J Gerontol A Biol Sci Med Sci, 71(8):1069-1073. 27091133 10.1093/gerona/glw053 PMC4945891

[84] Limke TL, Cai J, Miura T, Rao MS, Mattson MP (2003). Distinguishing features of progenitor cells in the late embryonic and adult hippocampus. Dev Neurosci, 25(2-4):257-272. 12966222 10.1159/000072273

[85] Chen X, Wu H, Chen H, Wang Q, Xie XJ, Shen J (2019). Astragaloside VI promotes neural stem cell proliferation and enhances neurological function recovery in transient cerebral ischemic injury via activating EGFR/MAPK signaling cascades. Mol Neurobiol, 56(4):3053-3067. 30088176 10.1007/s12035-018-1294-3

[86] Haiyan H, Rensong Y, Guoqin J, Xueli Z, Huaying X, Yanwu X (2016). Effect of Astragaloside IV on neural stem cell transplantation in Alzheimer’s disease rat models. Evid Based Complement Alternat Med, 2016:3106980. 27034688 10.1155/2016/3106980 PMC4806686

[87] Sun L, Han R, Guo F, Chen H, Wang W, Chen Z, et al. (2020). Antagonistic effects of IL-17 and Astragaloside IV on cortical neurogenesis and cognitive behavior after stroke in adult mice through Akt/GSK-3β pathway. Cell Death Discov, 6(1):1-18. 10.1038/s41420-020-00298-8 PMC7417740 32818074

[88] Bernardes de Jesus B, Schneeberger K, Vera E, Tejera A, Harley CB, Blasco MA (2011). The telomerase activator TA-65 elongates short telomeres and increases health span of adult/old mice without increasing cancer incidence. Aging Cell, 10(4):604-621. 21426483 10.1111/j.1474-9726.2011.00700.x PMC3627294

[89] Le Saux CJ, Davy P, Brampton C, Ahuja SS, Fauce S, Shivshankar P, et al. (2013). A novel telomerase activator suppresses lung damage in a murine model of idiopathic pulmonary fibrosis. PLoS One, 8(3):e58423. 23516479 10.1371/journal.pone.0058423 PMC3597721

[90] Yung LY, Lam WS, Ho MK, Hu Y, Ip FC, Pang H, et al. (2012). Astragaloside IV and cycloastragenol stimulate the phosphorylation of extracellular signal-regulated protein kinase in multiple cell types. Planta Med, 78(02):115-121. 22083896 10.1055/s-0031-1280346

[91] Gao H, Dou L, Shan L, Sun Y, Li W (2018). Proliferation and committed differentiation into dopamine neurons of neural stem cells induced by the active ingredients of radix astragali. Neuroreport, 29(7):577. 29481521 10.1097/WNR.0000000000000997 PMC6023595

[92] Zhang X, Chen J (2013). The mechanism of astragaloside IV promoting sciatic nerve regeneration. Neural Regen Res, 8(24):2256. 25206535 10.3969/j.issn.1673-5374.2013.24.005 PMC4146037

[93] Sun L, Zhang H, Wang W, Chen Z, Wang S, Li J, et al. (2020). Astragaloside IV exerts cognitive benefits and promotes hippocampal neurogenesis in stroke mice by downregulating interleukin-17 expression via wnt pathway. Front Pharmacol, 11:421. 32317974 10.3389/fphar.2020.00421 PMC7147333

[94] Cheng CY, Yao CH, Liu BS, Liu CJ, Chen GW, Chen YS (2006). The role of astragaloside in regeneration of the peripheral nerve system. J Biomed Mater Res A, 76(3):463-469. 16315188 10.1002/jbm.a.30249

[95] Tohda C, Tamura T, Matsuyama S, Komatsu K (2006). Promotion of axonal maturation and prevention of memory loss in mice by extracts of Astragalus mongholicus. Br J Pharmacol, 149(5):532-541. 16981006 10.1038/sj.bjp.0706865 PMC2014665

[96] Harris TE, Lawrence JC (2003). TOR signaling. Sci STKE, 2003(212):re15-re15. 14668532 10.1126/stke.2122003re15

[97] Gal-Ben-Ari S, Kenney JW, Ounalla-Saad H, Taha E, David O, Levitan D, et al. (2012). Consolidation and translation regulation. Learn Mem, 19(9):410-422. 22904372 10.1101/lm.026849.112 PMC3418764

[98] Burket JA, Benson AD, Tang AH, Deutsch SI (2015). NMDA receptor activation regulates sociability by its effect on mTOR signaling activity. Prog Neuropsychopharmacol Biol Psychiatry, 60:60-65. 25703582 10.1016/j.pnpbp.2015.02.009 PMC5549784

[99] Garelick MG, Kennedy BK (2011). TOR on the brain. Exp Gerontol, 46(2-3):155-163. 20849946 10.1016/j.exger.2010.08.030 PMC3432286

[100] Gao S, Zhang S, Zhou H, Tao X, Ni Y, Pei D, et al. (2021). Role of mTOR-regulated autophagy in synaptic plasticity related proteins downregulation and the reference memory deficits induced by anesthesia/surgery in aged mice. Front Aging Neurosci, 16(13):628541. 10.3389/fnagi.2021.628541 PMC8085306 33935683

[101] Fabrizio P, Pozza F, Pletcher SD, Gendron CM, Longo VD (2001). Regulation of longevity and stress resistance by Sch9 in yeast. Science, 292(5515):288-290. 11292860 10.1126/science.1059497

[102] Zhang D, Xuan J, Zheng BB, Zhou YL, Lin Y, Wu YS, et al. (2017). Metformin improves functional recovery after spinal cord injury via autophagy flux stimulation. Mol Neurobiol, 54(5):3327-3341. 27167128 10.1007/s12035-016-9895-1

[103] Zhou KL, Zhou YF, Wu K, Tian NF, Wu YS, Wang YL, et al. (2015). Stimulation of autophagy promotes functional recovery in diabetic rats with spinal cord injury. Sci Rep, 5(1):1-15. 10.1038/srep17130 PMC4657088 26597839

[104] Machado-Pereira M, Santos T, Ferreira L, Bernardino L, Ferreira R (2017). Anti-inflammatory strategy for M2 microglial polarization using retinoic acid-loaded nanoparticles. Mediators Inflamm, 2017:6742427. 29138531 10.1155/2017/6742427 PMC5613690

[105] Boland B, Kumar A, Lee S, Platt FM, Wegiel J, Yu WH, et al. (2008). Autophagy induction and autophagosome clearance in neurons: relationship to autophagic pathology in Alzheimer's disease. J Neurosci, 28(27):6926-6937. 18596167 10.1523/JNEUROSCI.0800-08.2008 PMC2676733

[106] Byles V, Covarrubias AJ, Ben-Sahra I, Lamming DW, Sabatini DM, Manning BD, et al. (2013). The TSC-mTOR pathway regulates macrophage polarization. Nat Commun, 4(1):1-11. 10.1038/ncomms3834 PMC3876736 24280772

[107] Laplante M, Sabatini DM (2012). mTOR signaling in growth control and disease. Cell, 149(2):274-293. 22500797 10.1016/j.cell.2012.03.017 PMC3331679

[108] Hoeffer CA, Klann E (2010). mTOR signaling: at the crossroads of plasticity, memory and disease. Trends Neurosci, 33(2):67-75. 19963289 10.1016/j.tins.2009.11.003 PMC2821969

[109] Triplett JC, Tramutola A, Swomley A, Kirk J, Grimes K, Lewis K, et al. (2015). Age-related changes in the proteostasis network in the brain of the naked mole-rat: implications promoting healthy longevity. Biochim Biophys Acta Mol Basis Dis, 1852(10):2213-2224. 10.1016/j.bbadis.2015.08.002 PMC4845741 26248058

[110] Perluigi M, Di Domenico F, Butterfield DA (2015). mTOR signaling in aging and neurodegeneration: at the crossroad between metabolism dysfunction and impairment of autophagy. Neurobiol Dis, 84:39-49. 25796566 10.1016/j.nbd.2015.03.014

[111] Caccamo A, Majumder S, Richardson A, Strong R, Oddo S (2010). Molecular interplay between mammalian target of rapamycin (mTOR), amyloid-β, and Tau: effects on cognitive impairments. J Biol Chem, 285(17):13107-13120. 20178983 10.1074/jbc.M110.100420 PMC2857107

[112] Spilman P, Podlutskaya N, Hart MJ, Debnath J, Gorostiza O, Bredesen D, et al. (2010). Inhibition of mTOR by rapamycin abolishes cognitive deficits and reduces amyloid-β levels in a mouse model of Alzheimer's disease. PloS One, 5(4):e9979. 20376313 10.1371/journal.pone.0009979 PMC2848616

[113] Lin J, Pan X, Huang C, Gu M, Chen X, Zheng X, et al. (2020). Dual regulation of microglia and neurons by Astragaloside IV-mediated mTORC1 suppression promotes functional recovery after acute spinal cord injury. J Cell Mol Med, 24(1):671-685. 31675186 10.1111/jcmm.14776 PMC6933381

[114] Stavoe AKH, Holzbaur ELF (2020). Neuronal autophagy declines substantially with age and is rescued by overexpression of WIPI2. Autophagy, 16(2):371-372. 31794336 10.1080/15548627.2019.1695401 PMC6984449

[115] Godbout JP, Johnson RW (2004). Interleukin-6 in the aging brain. J Neuroimmunol, 147(1-2):141-144. 14741447 10.1016/j.jneuroim.2003.10.031

[116] Zhang X, Liang T, Yang W, Zhang L, Wu S, Yan C, et al. (2020). *Astragalus membranaceus* injection suppresses production of interleukin-6 by activating autophagy through the AMPK-mTOR pathway in lipopolysaccharide-stimulated macrophages. Oxid Med Cell Longev, 2020:1364147. 32724488 10.1155/2020/1364147 PMC7364262

[117] Yang L, Dong X, Zhang W (2020). Astragaloside IV alleviates the brain damage induced by subarachnoid hemorrhage via PI3K/Akt signaling pathway. Neurosci Lett, 735:135227. 32619654 10.1016/j.neulet.2020.135227

[118] Cornu M, Albert V, Hall MN (2013). mTOR in aging, metabolism, and cancer. Curr Opin Genet Dev, 23(1):53-62. 23317514 10.1016/j.gde.2012.12.005

[119] Bordone L, Guarente L (2005). Calorie restriction, SIRT1 and metabolism: understanding longevity. Nat Rev Mol Cell Biol, 6(4):298-305. 15768047 10.1038/nrm1616

[120] Chung S, Yao H, Caito S, Hwang J-w, Arunachalam G, Rahman I (2010). Regulation of SIRT1 in cellular functions: role of polyphenols. Arch Biochem Biophys, 501(1):79-90. 20450879 10.1016/j.abb.2010.05.003 PMC2930135

[121] Haigis MC, Deng CX, Finley LW, Kim HS, Gius D (2012). SIRT3 is a mitochondrial tumor suppressor: a scientific tale that connects aberrant cellular ROS, the Warburg effect, and carcinogenesis. Cancer Res, 72(10):2468-2472. 22589271 10.1158/0008-5472.CAN-11-3633 PMC3354726

[122] Li M, Li SS, Dou BK, Zou YX, Han HZ, Liu DX, et al. (2020). Cycloastragenol upregulates SIRT1 expression, attenuates apoptosis and suppresses neuroinflammation after brain ischemia. Acta Pharmacol Sin, 41(8):1025-1032. 32203080 10.1038/s41401-020-0386-6 PMC7471431

[123] Shi YH, Zhang XL, Ying PJ, Wu ZQ, Lin LL, Chen W, et al. (2021). Neuroprotective effect of astragaloside IV on cerebral ischemia/reperfusion injury rats through Sirt1/Mapt pathway. Front Pharmacol, 12:6398898. 10.3389/fphar.2021.639898 PMC8033022 33841157

[124] Gu C, Zeng Y, Tang Z, Wang C, He Y, Feng X, et al. (2015). Astragalus polysaccharides affect insulin resistance by regulating the hepatic SIRT1-PGC-1α/PPARα-FGF21 signaling pathway in male Sprague Dawley rats undergoing catch-up growth. Mol Med Rep, 12(5):6451-6460. 26323321 10.3892/mmr.2015.4245 PMC4626146

[125] Mäkelä J, Tselykh TV, Maiorana F, Eriksson O, Do HT, Mudò G, et al. (2014). Fibroblast growth factor-21 enhances mitochondrial functions and increases the activity of PGC-1α in human dopaminergic neurons via Sirtuin-1. SpringerPlus, 3(1):2. 25932355 10.1186/2193-1801-3-2 PMC4409609

[126] Kullmann S, Heni M, Hallschmid M, Fritsche A, Preissl H, Häring H-U (2016). Brain insulin resistance at the crossroads of metabolic and cognitive disorders in humans. Physiol Rev, 96(4):1169-1209. 27489306 10.1152/physrev.00032.2015

[127] Akintola AA, van Heemst D (2015). Insulin, aging, and the brain: mechanisms and implications. Front Endocrinol, 6:13. 10.3389/fendo.2015.00013 PMC4319489 25705204

[128] Diehl T, Mullins R, Kapogiannis D (2017). Insulin resistance in Alzheimer's disease. Trans Res, 183:26-40. 10.1016/j.trsl.2016.12.005 PMC5393926 28034760

[129] Gheni G, Ogura M, Iwasaki M, Yokoi N, Minami K, Nakayama Y, et al. (2014). Glutamate acts as a key signal linking glucose metabolism to incretin/cAMP action to amplify insulin secretion. Cell Rep, 9(2):661-673. 25373904 10.1016/j.celrep.2014.09.030 PMC4536302

[130] Rothman SM, Olney JW (1995). Excitotoxicity and the NMDA receptor—still lethal after eight years. Trends Neurosci, 18(2):57-58. 7537407 10.1016/0166-2236(95)93869-y

[131] Yildiz-Unal A, Korulu S, Karabay A (2015). Neuroprotective strategies against calpain-mediated neurodegeneration. Neuropsychiatr Dis Treat, 11:297. 25709452 10.2147/NDT.S78226 PMC4327398

[132] Uttara B, Singh AV, Zamboni P, Mahajan R (2009). Oxidative stress and neurodegenerative diseases: a review of upstream and downstream antioxidant therapeutic options. Curr Neuropharmacol, 7(1):65-74. 19721819 10.2174/157015909787602823 PMC2724665

[133] Chiu BY, Chang CP, Lin JW, Yu JS, Liu WP, Hsu YC, et al. (2014). Beneficial effect of astragalosides on stroke condition using PC12 cells under oxygen glucose deprivation and reperfusion. Cell Mol Neurobiol, 34(6):825-837. 24807460 10.1007/s10571-014-0059-4 PMC11488913

[134] Yue R, Li X, Chen B, Zhao J, He W, Yuan H, et al. (2015). Astragaloside IV attenuates glutamate-induced neurotoxicity in PC12 cells through Raf-MEK-ERK pathway. PLoS One, 10(5):e0126603. 25961569 10.1371/journal.pone.0126603 PMC4427284

[135] Zhou X, Wang LL, Tang WJ, Tang B (2021). Astragaloside IV inhibits protein tyrosine phosphatase 1B and improves insulin resistance in insulin-resistant HepG2 cells and triglyceride accumulation in oleic acid (OA)-treated HepG2 cells. J Ethnopharmacol, 268:113556. 33157223 10.1016/j.jep.2020.113556

[136] Hanbing L, Jing N, Yunxue P, editors. Astragaloside IV improved insulin resistance in L6 myotubes induced by high glucose and insulin. 2011 International Conference on Remote Sensing, Environment and Transportation Engineering; 2011 24-26 June 2011:7415-7418.

[137] Grahame Hardie D (2014). AMP-activated protein kinase: a key regulator of energy balance with many roles in human disease. J Intern Med, 276(6):543-559. 24824502 10.1111/joim.12268 PMC5705060

[138] Salminen A, Kaarniranta K (2012). AMP-activated protein kinase (AMPK) controls the aging process via an integrated signaling network. Ageing Res Rev, 11(2):230-241. 22186033 10.1016/j.arr.2011.12.005

[139] Leverve X, Guigas B, Detaille D, Batandier C, Koceir E, Chauvin C, et al. (2003). Mitochondrial metabolism and type-2 diabetes: a specific target of metformin. Diabetes Metab, 29(4):6S88-6S94. 14502105 10.1016/s1262-3636(03)72792-x

[140] Wang C, Li Y, Hao M, Li W (2018). Astragaloside IV inhibits triglyceride accumulation in insulin-resistant HepG2 cells via AMPK-induced SREBP-1c phosphorylation. Front Pharmacol, 9:345-345. 29713279 10.3389/fphar.2018.00345 PMC5911465

[141] Wang BZ, Yang JJ, Zhang H, Smith CA, Jin K (2019). AMPK signaling regulates the age-related decline of hippocampal neurogenesis. Aging Dis, 10(5):1058-1074. 31595203 10.14336/AD.2019.0102 PMC6764723

[142] Becker L, Nguyen L, Gill J, Kulkarni S, Pasricha PJ, Habtezion A (2018). Age-dependent shift in macrophage polarisation causes inflammation-mediated degeneration of enteric nervous system. Gut, 67(5):827-836. 28228489 10.1136/gutjnl-2016-312940 PMC5565713

[143] Xu F, Cui W-Q, Wei Y, Cui J, Qiu J, Hu L-L, et al. (2018). Astragaloside IV inhibits lung cancer progression and metastasis by modulating macrophage polarization through AMPK signaling. J Exp Clin Cancer Res, 37(1):207. 30157903 10.1186/s13046-018-0878-0 PMC6116548

[144] Li L, Gan H, Jin H, Fang Y, Yang Y, Zhang J, et al. (2021). Astragaloside IV promotes microglia/macrophages M2 polarization and enhances neurogenesis and angiogenesis through PPARγ pathway after cerebral ischemia/reperfusion injury in rats. Int Immunopharmacol, 92:107335. 33429332 10.1016/j.intimp.2020.107335

[145] Sadria M, Layton AT (2020). Interactions among mTORC, AMPK, and SIRT: A Computational Model for Cell Energy Balance and Metabolism. Cell Commun Signal, 19(1):57. 10.1186/s12964-021-00706-1 PMC8135154 34016143

[146] Lee S, Kang S, Ang MJ, Kim J, Kim JC, Kim SH, et al. (2019). Deficiency of sterol regulatory element-binding protein-1c induces schizophrenia-like behavior in mice. Genes Brain Behav, 18(4):e12540. 30430717 10.1111/gbb.12540

[147] Zhou B, Zhou DL, Wei XH, Zhong RY, Xu J, Sun L (2017). Astragaloside IV attenuates free fatty acid-induced ER stress and lipid accumulation in hepatocytes via AMPK activation. Acta Pharmacol Sin, 38(7):998-1008. 28344322 10.1038/aps.2016.175 PMC5519246

[148] Xia X, Jiang Q, McDermott J, Han J-DJ (2018). Aging and Alzheimer's disease: comparison and associations from molecular to system level. Aging Cell, 17(5):e12802-e12802. 29963744 10.1111/acel.12802 PMC6156542

[149] Bettens K, Sleegers K, Van Broeckhoven C (2013). Genetic insights in Alzheimer's disease. Lancet Neurol, 12(1):92-104. 23237904 10.1016/S1474-4422(12)70259-4

[150] Selkoe DJ (2011). Resolving controversies on the path to Alzheimer's therapeutics. Nat Med, 17(9):1060-1065. 21900936 10.1038/nm.2460

[151] Chang CP, Liu YF, Lin HJ, Hsu CC, Cheng BC, Liu WP, et al. (2016). Beneficial effect of astragaloside on Alzheimer’s disease condition using cultured primary cortical cells under β-amyloid exposure. Mol Neurobiol, 53(10):7329-7340. 26696494 10.1007/s12035-015-9623-2

[152] Wang X, Xu W, Chen H, Li W, Li W, Zhu G (2020). Astragaloside IV prevents Aβ1-42 oligomers-induced memory impairment and hippocampal cell apoptosis by promoting PPARγ/BDNF signaling pathway. Brain Res, 1747:147041. 32739157 10.1016/j.brainres.2020.147041

[153] Wang X, Wang Y, Hu JP, Yu S, Li BK, Cui Y, et al. (2017). Astragaloside IV, a natural PPARγ agonist, reduces Aβ production in Alzheimer's disease through inhibition of BACE1. Mol Neurobiol, 54(4):2939-2949. 27023226 10.1007/s12035-016-9874-6

[154] Zhou F, Wang D (2017). The associations between the MAPT polymorphisms and Alzheimer's disease risk: a meta-analysis. Oncotarget, 8(26):43506-43520. 28415654 10.18632/oncotarget.16490 PMC5522165

[155] Idan-Feldman A, Ostritsky R, Gozes I (2012). Tau and caspase 3 as targets for neuroprotection. Int J Alzheimers Dis, 2012:493670. 22693678 10.1155/2012/493670 PMC3369463

[156] Puri BK, Morris G (2018). Potential therapeutic interventions based on the role of the endoplasmic reticulum stress response in progressive neurodegenerative diseases. Neural Regen Res, 13(11):1887. 30233059 10.4103/1673-5374.238614 PMC6183042

[157] Xia L, Guo D, Chen B (2017). Neuroprotective effects of astragaloside IV on Parkinson disease models of mice and primary astrocytes. Exp Ther Med, 14(6):5569-5575. 29285094 10.3892/etm.2017.5238 PMC5740776

[158] Ge B, Li S-l, Li F-r (2020). Astragaloside-IV regulates endoplasmic reticulum stress-mediated neuronal apoptosis in a murine model of Parkinson's disease via the lincRNA-p21/CHOP pathway. Exp Mol Pathol, 115:104478. 32511947 10.1016/j.yexmp.2020.104478

[159] Jiangbo Z, Xuying W, Yuping Z, Xili M, Yiwen Z, Tianbao Z (2009). Effect of astragaloside IV on the embryo-fetal development of Sprague-Dawley rats and New Zealand White rabbits. J Appl Toxicol, 29(5):381-385. 19367606 10.1002/jat.1422

[160] Wan X, Zhu J, Zhu Y, Ma X, Zheng Y, Zhang T, et al. (2010). Effect of astragaloside IV on the general and peripartum reproductive toxicity in Sprague-Dawley rats. Int J Toxicol, 29(5):505-516. 20884860 10.1177/1091581810376840

[161] Cho SJ, Yun SM, Jo C, Jeong J, Park MH, Han C, et al. (2019). Altered expression of Notch1 in Alzheimer's disease. PLoS One, 14(11):e0224941. 31770379 10.1371/journal.pone.0224941 PMC6879159

[162] Liu P, Zhao H, Luo Y (2017). Anti-aging implications of *Astragalus membranaceus* (Huangqi): a well-known chinese tonic. Aging Dis, 8(6):868-886. 29344421 10.14336/AD.2017.0816 PMC5758356

[163] Yu Y, Zhou L, Yang Y, Liu Y (2018). Cycloastragenol: an exciting novel candidate for age-associated diseases. Exp Ther Med, 16(3):2175-2182. 30186456 10.3892/etm.2018.6501 PMC6122403

[164] Wang F, Qian H, Kong L, Wang W, Wang X, Xu Z, et al. (2021). Accelerated bone regeneration by astragaloside IV through stimulating the coupling of osteogenesis and angiogenesis. Int J Biol Sci, 17(7):1821-1836. 33994865 10.7150/ijbs.57681 PMC8120474

[165] Gu Y, Wang G, Pan G, Fawcett JP, Sun J (2004). Transport and bioavailability studies of astragaloside IV, an active ingredient in *Radix astragali*. Basic Clin Pharmacol Toxicol, 95(6):295-298. 15569275 10.1111/j.1742-7843.2004.t01-1-pto950508.x

[166] Vinod C, Jena S (2021). Nano-neurotheranostics: impact of nanoparticles on neural dysfunctions and strategies to reduce toxicity for improved efficacy. Front Pharmacol, 12:612692. 33841144 10.3389/fphar.2021.612692 PMC8033012

[167] Lai WF (2022). Non-aromatic clusteroluminogenic polymers: structural design and applications in bioactive agent delivery. Mater Today Chem, 23:100712.

[168] Obireddy SR, Subbarao SMC, Venkata KRKS, Lai WF (2021). Development and characterization of montmorillonite-based hybrid materials for pH-responsive drug delivery. Chemistryselect, 6(7):1466-1470.

[169] Li CX, Obireddy SR, Lai WF (2021). Preparation and use of nanogels as carriers of drugs. Drug Deliv, 28(1):1594-1602. 34308729 10.1080/10717544.2021.1955042 PMC8317930

[170] Lai WF, Lin MC (2015). Folate-conjugated chitosan-poly(ethylenimine) copolymer as an efficient and safe vector for gene delivery in cancer cells. Curr Gene Ther, 15(5):472-480. 26264706 10.2174/1566523215666150812120347

[171] Lai WF, Tang GP, Wang X, Li G, Yao H, Shen Z, et al. (2011). Cyclodextrin-PEI-Tat polymer as a vector for plasmid DNA delivery to placenta mesenchymal stem cells. Bionanoscience, 1(3):89-96. 23024930 10.1007/s12668-011-0010-9 PMC3460531

[172] Wang K, Li L, Xu X, Lu L, Wang J, Wang S, et al. (2019). Fe_3_O_4_@astragalus polysaccharide core-shell nanoparticles for iron deficiency anemia therapy and magnetic resonance imaging in vivo. ACS Appl Mater Interfaces, 11(11):10452-10461. 30801182 10.1021/acsami.8b18648

[173] Wang Y, Fan X, Qu H, Gao X, Cheng Y (2012). Strategies and techniques for multi-component drug design from medicinal herbs and traditional Chinese medicine. Curr Top Med Chem, 12(12):1356-1362. 22690682 10.2174/156802612801319034

[174] Pan R, Zhou M, Zhong Y, Xie J, Ling S, Tang X, et al. (2019). The combination of Astragalus membranaceus extract and ligustrazine to improve the inflammation in rats with thrombolytic cerebral ischemia. Int J Immunopathol Pharmacol, 33:2058738419869055. 31409163 10.1177/2058738419869055 PMC6696830

[175] Chen YS, Chang SS, Ng HY, Huang YX, Chen CC, Shie MY (2021). Additive manufacturing of astragaloside-containing polyurethane nerve conduits influenced Schwann cell inflammation and regeneration. Processes, 9(2):353.

[176] Sun B, Rui R, Pan H, Zhang L, Wang X (2018). Effect of combined use of astragaloside IV (AsIV) and atorvastatin (AV) on expression of PPAR-γ and inflammation-associated cytokines in atherosclerosis rats. Med Sci Monit, 24:6229-6236. 30190450 10.12659/MSM.908480 PMC6139110

[177] Lai WF, Huang E, Lui KH (2021). Alginate-based complex fibers with the Janus morphology for controlled release of co-delivered drugs. Asian J Pharm Sci, 16(1):77-85. 33613731 10.1016/j.ajps.2020.05.003 PMC7878464

[178] Lai WF, Rogach AL, Wong WT (2018). One-pot synthesis of an emulsion-templated hydrogel-microsphere composite with tunable properties. Compos Part A-Appl S, 113:318-329.

[179] Lai WF, Susha AS, Rogach AL (2016). Multicompartment microgel beads for co-delivery of multiple drugs at individual release rates. ACS Appl Mater Interfaces, 8(1):871-880. 26720613 10.1021/acsami.5b10274

[180] Mukonzo J, Aklillu E, Marconi V, Schinazi RF (2019). Potential drug-drug interactions between antiretroviral therapy and treatment regimens for multi-drug resistant tuberculosis: implications for HIV care of MDR-TB co-infected individuals. Int J Infect Dis, 83:98-101. 30991140 10.1016/j.ijid.2019.04.009 PMC7700887

[181] Kalombo L, Lemmer Y, Semete-Makokotlela B, Ramalapa B, Nkuna P, Booysen LLLIJ, et al. (2019). Spray-Dried, nanoencapsulated, multi-drug anti-tuberculosis therapy aimed at once weekly administration for the duration of treatment. Nanomaterials, 9(8):1167. 10.3390/nano9081167 PMC6724112 31443150

[182] Zhang J, Wu C, Gao L, Du G, Qin X (2020). Astragaloside IV derived from Astragalus membranaceus: A research review on the pharmacological effects. Adv Pharmacol, 87:89-112. 32089240 10.1016/bs.apha.2019.08.002

[183] Lai WF, Gui DY, Wong M, Doring A, Rogach AL, He TC, et al. (2021). A self-indicating cellulose-based gel with tunable performance for bioactive agent delivery. J Drug Deliv Sci Tec, 63:102428.

